# Progress toward implementing the Swiss Hepatitis Strategy: Is HCV elimination possible by 2030?

**DOI:** 10.1371/journal.pone.0209374

**Published:** 2018-12-31

**Authors:** Beat Müllhaupt, Philip Bruggmann, Florian Bihl, Sarah Blach, Daniel Lavanchy, Homie Razavi, Sarah Robbins Scott, David Semela, Francesco Negro

**Affiliations:** 1 Swiss HPB (Hepato-Pancreato-Biliary) Center and Department of Gastroenterology and Hepatology, University Hospital Zürich, Zürich, Switzerland; 2 Arud Centres for Addiction Medicine, Zürich, Switzerland; 3 Gastroenterology Department, Ospedale Cantonale, Bellinzona, Switzerland; 4 Center for Disease Analysis (CDA), Lafayette, Colorado, United States of America; 5 Consultant, Ruelle des Chataigniers 1, Denges VD, Switzerland; 6 Division of Gastroenterology & Hepatology, Cantonal Hospital St. Gallen, St. Gallen, Switzerland; 7 Divisions of Gastroenterology and Hepatology and of Clinical Pathology, University Hospital, Genève, Switzerland; Centers for Disease Control and Prevention, UNITED STATES

## Abstract

Catalyzed by the concerns over the growing public health and economic burden of Hepatitis C virus (HCV) in Switzerland, a diverse group of experts and patient representatives came together in 2014 to develop the Swiss Hepatitis Strategy, setting targets for the elimination of viral hepatitis in Switzerland by 2030. Previous studies have reported the estimated number of chronic HCV infections and forecasted burden of disease given different intervention strategies. However, given new prevalence data by the Swiss Federal Office of Public Health, which decreased total infections by about half, an updated analysis is warranted. We aimed to provide an updated viremic prevalence estimate for Switzerland and evaluate the impact on forecasted liver related morbidity and mortality of an ‘inaction’ scenario and intervention scenarios to achieve the Global Health Sector Strategy for Viral Hepatitis and Swiss Hepatitis Strategy goals by 2030. A Markov disease-progression model was used to calculate the present and future burden of HCV infection by disease stage according to these different strategies. In 2017, there were an estimated 36,800 (95% UI: 26,900–39,200) viremic infections in Switzerland. Given the current standard of care, total viremic infections are expected to decline by 45%, while cases of decompensated cirrhosis, hepatocellular carcinoma, and liver-related deaths will decrease by 20%. If treatment and diagnosis efforts were to cease in 2018, late stage HCV-related morbidity and mortality would increase by 90–100% by 2030. Increasing treatment and diagnosis to achieve the Global Health Sector Strategy or Swiss Hepatitis Strategy goals by 2030, will reduce the number of chronic infections to less than 13,000 and 4,000, respectively. Although the HCV epidemic is declining in Switzerland, efforts to expand diagnosis and treatment are needed to achieve elimination by 2030.

## Introduction

In 2014, we conducted a study to expand modeling efforts and assist in the development of a national strategy for hepatitis C virus (HCV) control in Switzerland [1]. In the absence of a representative, recent prevalence estimate, the analysis used a base anti-HCV prevalence of 1.6% (range: 0.8%– 1.8%) in 1998 [2–4]. Assuming a 79.7% viremic rate [5], this resulted in a viremic prevalence of approximately 1.3% in 1998 and a 1.0% viremic prevalence in 2015, corresponding to 78,000 active HCV infections [6].

In 2015, the Swiss Federal Office of Public Health (FOPH) commissioned a situational analysis to inform programmatic and planning efforts to control viral hepatitis in Switzerland. Results published in 2017, estimated 36,000–43,000 HCV-RNA positive cases in 2016, corresponding to a 0.45%– 0.54% viremic prevalence, after accounting for mortality and cured patients [7].

In light of this new information, our disease burden model was revisited, incorporating updated data on HCV diagnosis, treatment and sustained viral response (SVR) from 2014 through 2017. The outcome is an up-to-date forecast of prevalence and end-stage outcomes associated with HCV. Additionally, this analysis seeks to highlight the gains that have been made since the formulation and launching of the Swiss Hepatitis Strategy (SHS) initiative in 2014 [8]. The aim of this initiative is to achieve a reduction in new infections, total viremic infections, liver transplants, and cases of hepatocellular carcinoma (HCC) of 30% by 2020 and 90% by 2030 [8]. Authors also tracked Switzerland’s progress toward achieving the Global Health Sector Strategy (GHSS) targets for the elimination of HCV as a public health threat by 2030 of a 65% reduction in mortality; 90% diagnosis coverage of the infected population; and a 90% reduction in new infections [9] (S1 Table).

## Methodology

A previously described disease burden model [1, 10] seeded with Swiss-specific population estimates, mortality and HCV epidemiology data was calibrated to forecast the present and future burden of viremic infections as well as viremic infections by disease sequelae (S1 File). There were estimated to be 36,000–43,000 (midpoint 39,500) HCV-RNA positive (viremic) cases alive in Switzerland by the end of 2016 [7].

The number of individuals previously diagnosed with HCV as of 2015 was calculated by birth year and sex for five-year reporting periods between 1988 and 2015 using FOPH notification data. Notification data after 2015 were available at an aggregate level, but not stratified by age and sex, and were considered separately. Between 1988 and 2015, more than 48,400 cases of HCV were reported by age and sex [11]. Reported cases were adjusted once for viremia (79.7% viremic) [5] and then annually for age-specific all-cause and liver-related mortality. Cured cases were additionally removed from the HCV-infected pool on an annual basis. After the adjustments were made, each year, the “surviving” cases were aged to the next year, and the process for removing mortality and cured cases was repeated. Following this process, of the total 36,000–43,000 estimated viremic infected individuals in the entire population, there were estimated to be between 25,500 and 29,100 (midpoint 25,800) viremic (chronic, RNA-positive infections) diagnosed cases still alive at the end of 2015 (62%-71% of total prevalent cases in 2015). According to aggregate notification data, after 2015 an additional 1,116 viremic cases were newly diagnosed each year. New infections were estimated by the model accounting for spontaneous clearance and cure (S1 File) [6, 12]. Liver cancer data reported to National Institute for Cancer Epidemiology and Registration (NICER) from 1999 to 2013 were adjusted for HCV-attributable HCC cases based on histology data from the Geneva Tumor Registry [13], and were then used to validate modelled HCC trends (S1 File).

Based on extrapolations of data provided by the Swiss Pharmacist Cooperative (OFAC), the Swiss National Pharmacy Service (Mediservice) and IMS Health, approximately 3,000 patients initiated treatment in 2017, with an estimated average SVR of 95% for all genotypes [14, 15].

A “baseline” scenario was developed using the 2017 treatment paradigm, in which 1,116 viremic cases were diagnosed and 3,000 patients, with no fibrosis restriction (≥F0), were treated by the end of each year. To model the expected depletion of the pool of available patients [1], a trend was introduced, assuming the number of patient treated annually would reduce by half by 2020. The annual number of treated patients was held constant from 2020 through 2030.

Three intervention scenarios were developed to model the following treatment paradigms beginning in 2018: 1) “inaction” discontinue treatment (i.e. take no further action) to represent what may happen on an individual level; 2) increase the annual number of ≥F0 patients treated to achieve the SHS targets for HCV (30% reduction in new infections, total viremic infections, liver transplants, and HCC cases by 2020 and a 90% reduction by 2030); and 3) increase the annual number of ≥F0 patients treated to achieve the GHSS targets for HCV (90% reduction in new infections, 65% reduction in liver related mortality, and a 90% diagnosis rate among the infected population).

Sensitivity analyses were completed using Crystal Ball, an Excel add-in by Oracle. Beta-PERT distributions were used to model uncertainty associated with inputs, and Monte Carlo simulations were used to determine 95% uncertainty intervals (UIs).

## Results

**Fig 1:**
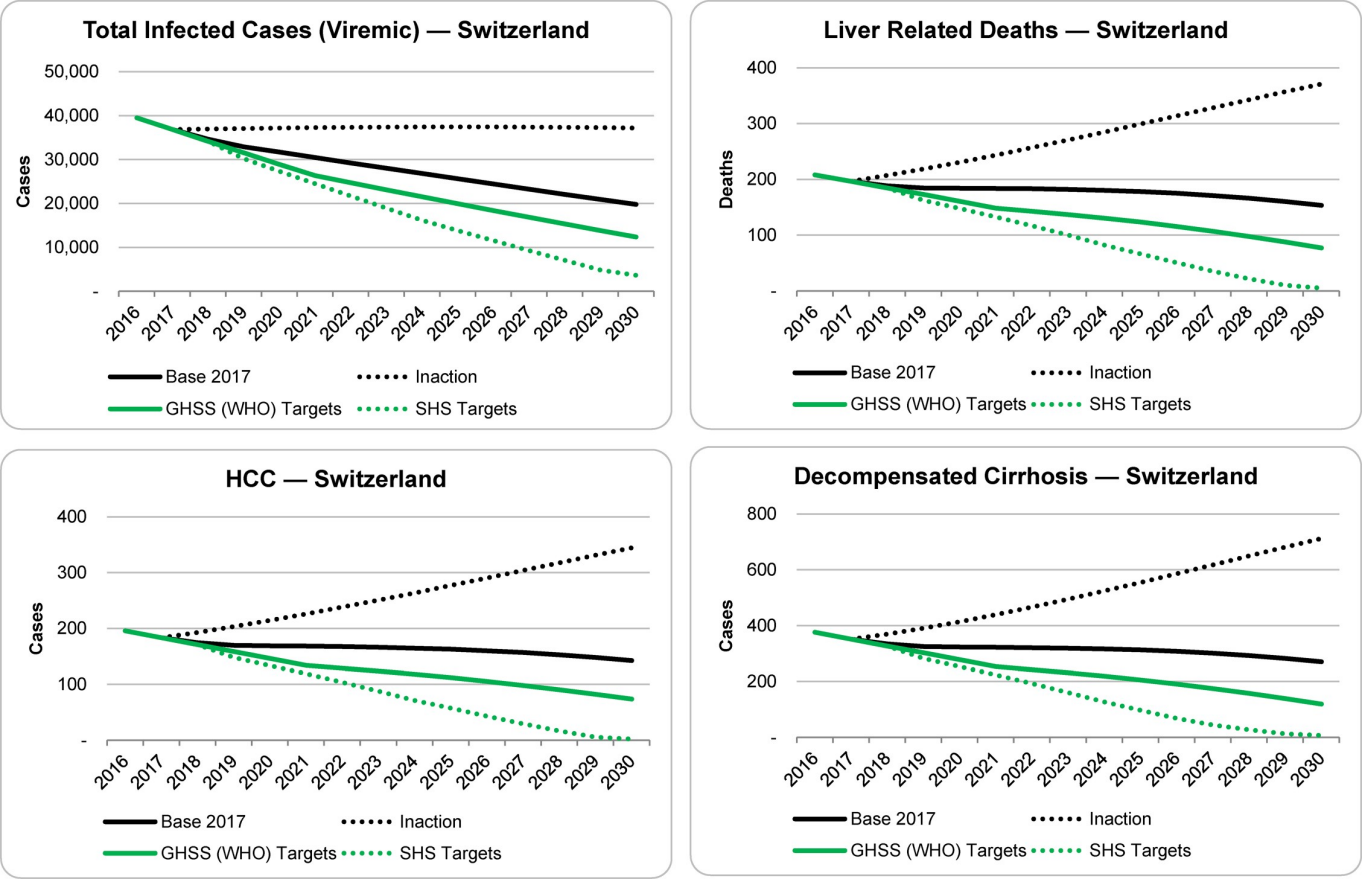
Projected outcomes for total viremic infections, liver related deaths, HCC and decompensated cirrhosis under the base, inaction and elimination scenarios, 2016–2030.

Under the base scenario, a total of 21,000 patients would be treated between 2018 and 2030 (Table 1). The total number of viremic infections is estimated to decrease from 39,500 (95% UI: 36,000–43,000) in 2016 to 36,800 (95% UI: 26,900–39,200) viremic infections in 2017, due to the increase in patients treated and cured. As shown in Fig 1 and Table 2, after considering deaths (all-cause and liver-related) and new infections, the total number of infected cases in 2030 was projected to be roughly half that of 2017, or approximately 19,800 (95% UI: 10,400–22,100) viremic infections in 2030. Meanwhile, end stage outcomes such as decompensated cirrhosis (DC), hepatocellular carcinoma (HCC) and liver-related deaths (LRD) were projected to decline by 20–25%, relative to 2017, with approximately 80, 40 and 50 fewer total cases and deaths, respectively, by 2030 (Table 2).

**Table 1:** Annual number diagnosed and initiating treatment, as well as treatment eligibility and SVR under the base, inaction and elimination scenarios, 2016–2025.

Scenario Input	Scenario	Annual							Cumulative
		2016	2017	2018	2019	2020	2022	≥2025	2018–2030
**Newly Diagnosed**	Base	1,100	1,100	1,100	1,100	1,100	1,100	1,100	14,500
	Inaction	1,100	1,100	-	-	-	-	-	0
	GHSS	1,100	1,100	1,100	1,100	1,500	1,500	1,500	18,700
	SHS	1,100	1,100	1,100	1,400	1,400	1,400	1,400	17,500
**Initiating Treatment**	Base	2,000	3,000	2,500	2,000	1,500	1,500	1,500	21,000
	Inaction	2,000	3,000	-	-	-	-	-	0
	GHSS	2,000	3,000	3,000	3,000	3,000	2,000	2,000	30,000
	SHS	2,000	3,000	3,000	4,400	3,300	3,300	2,900	40,300
**Treatment Eligibility, Fibrosis**	All scenarios	≥ F2	≥ F2	≥ F0	≥ F0	≥ F0	≥ F0	≥ F0	-
**Treatment Eligibility, Age (years)**	All scenarios	15+	15+	15+	15+	15+	15+	15+	-
**SVR**	All scenarios	95%	95%	95%	95%	95%	95%	95%	-

**Table 2:** Change in viremic infections, decompensated cirrhosis, hepatocellular carcinoma and liver related deaths between 2017–2030.

Outcome	Scenario	Prevalent Cases				Incident Cases		
		2017	2030	Absolute change*	Percent change**	2017	2030	Cases Averted***
**Viremic Infections**	Base	36,800	19,800	-17,000	-45%	700	^†^	^†^
	Inaction	36,800	37,100	300	1%			
	GHSS	36,800	12,300	-24,500	-65%			
	SHS	36,800	3,600	-33,200	-90%			
**Decompensated cirrhosis**	Base	350	270	-80	-25%	110	90	-
	Inaction	350	710	360	100%	110	200	-730
	GHSS	350	120	-230	-65%	110	50	320
	SHS	350	7	-340	-100%	110	5	630
**Hepatocellular carcinoma**	Base	180	140	-40	-20%	140	110	-
	Inaction	180	340	160	90%	140	250	-920
	GHSS	180	70	-110	-60%	140	60	400
	SHS	180	2	-180	-100%	140	6	790
**Liver related deaths**^‡^	Base	200	150	-50	-25%	200	150	-
	Inaction	200	370	170	85%	200	370	-1,400
	GHSS	200	80	-120	-60%	200	80	600
	SHS	200	5	-200	-100%	200	5	1,200

Values in this table have been rounded, so calculated percentages may not be reproducible

* Absolute change was calculated as the 2030 prevalent value minus the 2017 prevalent value

** Percent (%) change was calculated as the 2030 prevalent value divided by the 2017 prevalent value, minus one

*** Cases averted is calculated as cumulative incident infections from 2017 to 2030 under the base scenario, minus cumulative incident cases from 2017–2030 in the intervention scenario

^†^ Incidence of HCV was not modeled dynamically in this analysis

^‡^ Liver related deaths are an annual measure considered in both incident and prevalent sections here, for calculation purposes

In the inaction scenario, discontinuing treatment after 2017 would cause prevalence to increase slightly (37,100 cases remaining in 2030) due to continued mortality and occurrence of new cases; however, rates of DC, HCC and LRD would increase 90%– 100% as shown in Fig 1 and Table 2. Compared with the baseline scenario, discontinuing treatment would result in more incident cases of DC, HCC and LRD (730, 920 and 1,400 respectively) between 2017 and 2030.

The model was then used to calculate the threshold of diagnosed and treated patients needed to achieve both the GHSS and SHS goals. Achieving the GHSS Targets by 2030 would also require a sustained increase in the number of diagnosed and treated infected individuals. 1,500 patients would need to be diagnosed annually beginning in 2020 and a maximum of 3,000 patients would need to be treated annually from 2017–2021. Under this scenario, there would be an estimated 65% reduction in total viremic infections, leaving 12,300 cases by 2030 (Table 1). Incident cases of HCC and DC would be reduced by 60% and 65%, respectively, compared to the base scenario, with 320 and 400 cases averted by 2030. More than 600 LRD could be averted by achieving the GHSS Targets by 2030. Compared to the inaction scenario, there would be 1,000 fewer incident cases of DC, 1,300 fewer incident cases of HCC, and 2,000 LRD averted by 2030.

Meeting SHS elimination targets for HCV by 2030 would require diagnosing at least 1,400 new viremic cases per year beginning in 2019 and treating a maximum of 4,400 patients annually in 2019 (Table 1). In total, 40,300 patients would need to be treated between 2018 and 2030. This would result in a 90% decline in total infected cases by 2030, with approximately 3,600 cases remaining in 2030. Rates of DC, HCC and LRD were all projected to fall by 95–100% with 340, 180 and 195 fewer total cases, respectively, between 2017 and 2030. Compared to the base, this scenario showed 630 incident cases of DC, 790 incident cases of HCC and 1,200 LRD averted by 2030. Compared to the inaction scenario, this scenario showed 1,400 incident cases of DC, 1,710 incident cases of HCC and 2,600 LRD averted by 2030. Compared to the GHSS Targets scenario, this scenario showed 300 fewer incident cases of DC, 390 fewer incident cases of HCC and 2,570 more liver-related deaths averted by 2030.

### Sensitivity and uncertainty analyses, and model validation

In a sensitivity analysis, the progression rate from acute infection to spontaneous clearance and by the starting viremic prevalence in 2016 accounted for more than 85% of variance in 2017 viremic prevalence, combined (S2 File). If the 2016 viremic prevalence was at upper range of the FOPH estimate (43,000 instead of 39,500), we would expect the total number of viremic infections in 2017 to increase (38,000 rather than 36,000). In a separate sensitivity analysis which looked at projections of viremic infections in 2030, the number of patients treated annually between 2020 and 2030 accounted for more than 50% of explained variation. Reducing the annual number of patients treated under the baseline scenario (from 1,500 to 1,000 per year) would increase the forecasted number of viremic cases (from 20,000 to 23,000) remaining in 2030.

Model validation using NICER liver cancer data (S1 File) identified that HCC model projections are on the low range of reported cases (after adjusting reported NICER liver cancer cases for HCV-attribuable HCC). This can be explained by the current lower estimate of viremic prevalence used in the model. In order to calibrate the model to achieve the number of reported HCC cases, a 2016 viremic prevalence estimate of 0.8%, rather than 0.5%, would be needed. Consequently, if the viremic prevalence was 0.8% in 2016, almost double the number of patients would need to be diagnosed and treated over the next ten years in order to achieve both the GHSS and SHS goals. A summary of these outcomes can be found in S2 File.

## Discussion

Experts from across Switzerland collaborated to develop a national strategy for the elimination of hepatitis by 2030 [16]. These efforts have initiated an increase in research, analyses and forecasts, which require updates to our original modeling efforts. Updated findings were incorporated into a disease burden model to assess the impact of maintaining the status quo, inaction, or expanding treatment access to meet GHSS and SHS elimination targets by 2030.

Compared with original projections published in 2014, which showed a reduction in viremic cases but a 50%– 85% increase in end-stage liver disease (decompensated cirrhosis and HCC) and mortality by 2030 [1], the current analysis shows a reduction in all indicators by 2030. The current outcome is attributable to a lower starting prevalence, combined with enhanced efforts over the last four years to expand access to HCV treatment. Since 2015, more than 2,000 ≥F2 patients have been treated annually with direct acting antivirals (DAAs). Over the course of 2017, treatment restrictions were lifted such that by October, all patients could be treated, regardless of fibrosis stage.

Despite the recent successes managing HCV across Switzerland, the scenario showing outcomes related to discontinued treatment provides a sobering message that HCV prevention and treatment must remain a priority in order to reduce HCV-related morbidity and mortality. Although this scenario is unlikely to occur in the real world on a national scale (i.e. complete discontinuation of all diagnostic and treatment efforts), it does provide a “worst case” scenario and exposes what could happen on an individual or community level, should treatment stall.

Due to the removal of fibrosis restrictions in 2017 and an increase in the number of patients under care, Switzerland was listed as “on track” for achieving the GHSS 2030 elimination goals, as of early 2018. However, as seen in the GHSS scenario in Table 1, treatment must be sustained at a level of 3,000 patients annually through 2021 to maintain this success. Preliminary reports on treatment uptake for 2018 indicate that the number of patients initiating treatment each month is decreasing and will likely drop below the threshold for elimination in the coming year (IMS Health Data, personal communication). Thus, continued efforts will be necessary to maintain the momentum toward elimination targets.

Eliminating HCV through achieving the Swiss Hepatitis Strategy (SHS) targets is also possible by 2030 with sustained diagnosis of 1,400 patients and treatment of 4,400 patients annually in 2019. Given current model estimates, 60% of infected patients were diagnosed as of 2015. This is in line with other published studies which estimate around 40–85% of individuals are aware of their status [17]. Already in the last year, 1,116 new viremic patients were identified, suggesting that sustaining an increase in the number of patients linked to care is feasible. However, experience from outside of Switzerland has shown that even with a formal screening strategy, countries such as Egypt have struggled to sustain diagnostic and treatment efforts as the patient pool is depleted [18]. For example, if Switzerland was to discontinue diagnosing new patients in the upcoming year, the country could maintain treatment at current levels (1,500 per year) through 2032, by which time the eligible pool of linked-to-care patients would be depleted. Following this trend, neither the GHSS nor the SHS strategies could be achieved.

Using the calculations described by Bruggmann et al [19], and considering the prevalence and number of cases diagnosed under the base case above; in 2018, 130 individuals would need to be screened to diagnose one new viremic case [19]. By 2027, however, as prevalence drops, and patients become harder to find, this would increase to nearly 240 individuals screened per new case identified. The screening campaign would be more efficient if individuals testing negative for HCV are tracked in a registry, to avoid being screened twice. Under this scenario, the number needed to screen would begin to drop after 2027.

This analysis had a number of limitations. It assumed that there are already efforts in place to reduce new infections, including continuation of harm reduction programs and access to care in high risk populations. It also assumes that all diagnosed patients are available to treat. In reality, losses exist at each stage in the cascade of care [20]. Thus, efforts to proactively link diagnosed patients to care and simplify the cascade of care will be needed to make achieving the GHSS targets for 2030 a reality. The emergence of rapid RNA tests (as yet unapproved), non-invasive methods for liver disease staging, pan-genotypic therapies, and facilitated treatment access for patients of all fibrosis stages are welcome steps in this process, as it means that any patients identified can immediately be treated or referred to treatment [20]. The bottleneck existing at the linkage-to-care level could be widened by allowing general practitioners to treat HCV as has been done successfully in Australia [21]. More so, the model used is not dynamic; it does not take into consideration the effect of treatment as prevention or reinfection in the population. As such, all predictions are conservative in nature and fewer patients may need to be linked to care and treated in order to achieve both strategies.

In addition, the analysis suggests that the prevalence of HCV could be higher based on the modeled HCC estimates for 1990–2013 compared to incident liver cancer data from NICER. If the prevalence of HCV is indeed higher than estimated, the number of individuals needing to be diagnosed and treated will be higher (S2 File).

The analysis also suggests that the current risk-based testing strategy needs to be enhanced with additional strategies to find those infected with HCV. Birth cohort screening strategies, for example, have the potential to find previously undetected cases while being sufficiently cost-effective [22]. A recent study from France showed that universal screening is a cost-effective strategy for new cases identification [23]. Combined with a small increase in treatment, elimination of HCV by 2030, as defined by the GHSS and SHS, is achievable.

## Supporting information

S1 Table Comparison of the goals of the Swiss Hepatitis Strategy (SHS) and the Global Health Sector Strategy (GHSS). (DOCX)

S1 File HCV disease burden model, forecasting viremic prevalence. (DOCX)

S2 File Outcomes of the sensitivity and uncertainty analyses. (DOCX)
