# Supplementary material for: Progress toward implementing the Swiss Hepatitis Strategy: Is HCV elimination possible by 2030?
Source: PLoS One. 2018 Dec 31;13(12):e0209374. doi: 10.1371/journal.pone.0209374 (PMC6312389; doi:10.1371/journal.pone.0209374)
Supplement: S1 Table — (DOCX) [file pone.0209374.s001.docx]

# S1 Table. Comparison of the goals of the Swiss Hepatitis Strategy and the Global Health Sector Strategy.

|  | SHS | | GHSS |
| --- | --- | --- | --- |
|  | 2020 | 2030 | 2030 |
| Reduction of:  New infections  Total viremic infections  Diagnosis coverage  Liver transplants  HCC cases  Mortality | 30%  30%  NA  30%  30%  NA | 90%  90%  NA  90%  90%  NA | 90%  NA  90%  NA  NA  65% |

SHS, Swiss Hepatitis Strategy; GHSS, Global Health Sector Strategy; NA, not applicable
