# Supplementary material for: Progress toward implementing the Swiss Hepatitis Strategy: Is HCV elimination possible by 2030?
Source: PLoS One. 2018 Dec 31;13(12):e0209374. doi: 10.1371/journal.pone.0209374 (PMC6312389; doi:10.1371/journal.pone.0209374)
Supplement: S1 File — (DOCX) [file pone.0209374.s002.docx]

# S1 File. HCV disease burden model, forecasting viremic prevalence

**Indicator —** This analysis focused on estimating viremic HCV infections, which reflects the presence of HCV RNA. The analysis used anti-HCV prevalence, serological evidence of past or present infection, and the viremic rate in a Markov model to estimate the end-of-year viremic prevalence in 2017.

**Time period —** Available published and unpublished studies conducted before July 2018 were considered for model input values. Model outcomes concerning disease burden (including viremic prevalence, incidence and prevalence of HCC, decompensated cirrhosis and mortality) were forecast through 2030, in line with the Global Health Sector Strategy Targets and Swiss Hepatitis Strategy Targets. Health effects associated with these outcomes were assessed through 2030.

**Geographical scope —** The analysis was focused on the HCV-infected population residing in Switzerland, at a national level.

**Modeling HCV Prevalence**

The analysis started with a review of published studies and was supplemented with Delphi method interviews with country experts to ensure that all relevant data (including unpublished data) are taken into consideration. A Markov model was used to forecast HCV prevalence over time. The prevalence of HCV is not constant over time. When incident cases are higher than deaths and cures, the total number of infections will increase over time. The total number of infections will decrease over time when the opposite is true. The model was used to forecast HCV prevalence at the end of 2017. The details of the model have been published previously [1, 2].

**Required inputs —** The following inputs were required to build and calibrate the Swiss model.

| Model input | Definition | Source |
| --- | --- | --- |
| Country population by 5-year age group | Number of people in the Switzerland reported annually from 1950 to 2050 (by sex and 5-year age group) | [3] |
| Mortality rate by  5-year age group | Share of deaths among the total population, annually from 1950 to 2050 (by sex and 5-year age group) | [4] |
| Anti-HCV prevalence | Share of total population who are anti-HCV(+) | [5] |
| Viremic rate | Percent of anti-HCV(+) individuals who are HCV RNA(+) | [6] |
| Age and sex distribution | HCV prevalence by sex and 5-year age group | Calculated using diagnosis data from the Swiss FOPH, adjusted to the size of the prevalent population [7, 8] [5] |
| Genotype distribution | Proportion of HCV RNA(+) population categorized by HCV genotype (out of 100%) | [9] |
| Annually treated | Number of HCV-infected individuals who have received treatment in a given year | IMS Health [8]  Expert Input (Prof. Franco Negro) |
| Total diagnosed | Viremic HCV cases diagnosed and alive in a given year | Calculated using data from [7, 8] |
| Newly diagnosed | Annual number of newly diagnosed HCV cases | [7, 8] |
| Liver transplants | Annual number of liver transplantations due to HCV | Swiss Transplant; Personal communication |
| HCC | Annual incident cases of HCC due to HCV | NICER; Geneva Tumor Registry; [10] |

**Prevalence by age —** Switzerland’s notification data were used to estimate HCV prevalence by age. In this method, the annual number of newly diagnosed cases in Switzerland was collected and adjusted for mortality and cures. The birth year was used to calculate the age and consolidate data from multiple years into the last year of available data. It was assumed that screening was conducted randomly, and the number of diagnosed cases by each age group was divided by the country’s population in that age group (in the last year of data). A weighting factor was applied to get the sum-product of the rough prevalence by age and general populations by age equal to the estimated total infections in the country. This weighting factor times the rough prevalence was used as an estimate of the true prevalence by age. The output was approved by the expert panel. Diagnosed data by age were available through the Federal Office of Public.[11]

**Treated patients —** IMS data showed an estimated 2,300 (2,000–2,500) total patients treated in Switzerland in 2015, approximately 1,280 of whom were ≥F3 and the remainder of whom were F2 [8]. In 2016, the number of treated patients dropped to 2,100 ≥F2 (Table 1). As of October 2017, treatment restrictions were lifted, making patients of all fibrosis stages eligible for treatment. In 2017, 3,000 patients were treated (IMS Health data) — an increase of 43% compared to 2016 as previously warehoused F0 and F1 patients had begun to seek treatment.

**Cured patients —** In the absence of better information, it was assumed the genotype distribution of the treated population was the same as that of the total infected population (they have the same probability of being diagnosed and treated). The sustained virologic response (SVR) rates by genotype were used to estimate the number of patients cured per year. Country interviews were used to determine the real-world SVR for the different treatment regimens — interferon-based therapy in combination with ribavirin (RBV) (dual therapy), with RBV and a protease inhibitor (PI) (triple therapy), and RBV with direct-acting antivirals (DAAs). Experts took into consideration the percentage of the population that was treatment-experienced and treatment-naïve on each treatment option and disease stages of the patients being treated (e.g., F1, F2, F3 and F4). The average SVR by genotype by country has been reported previously [2, 12, 13]. SVR in countries without expert interviews were extrapolated from countries with interviews.

**Liver transplantations —** The annual number of liver transplantations was gathered from Swiss Transplant and adjusted for the percentage attributed to HCV infection based on expert consensus. For examples of such an adjustment factor, see references listed here [14-19].

**Diagnosed patients —** Notification data from 1988–2015 from the Swiss Federal Office of Public Health was utilized.[11] Diagnosed cases were calculated by summing data from all years after taking into consideration the mortality among the diagnosed cases. It was assumed that the viremic rate among the diagnosed population was the same as the total infected population.

**All-cause mortality —** The all-cause mortality rates by age and sex were gathered from the from the United Nations World Population Prospects [4]. The rates were adjusted for incremental increase in mortality due to injection drug use (IDU) and history of blood transfusion in the HCV-infected population. A standardized mortality ratio (SMR) of 10 (9.5–29.9) was used for the portion of the HCV-infected population that was active PWID aged 15–44 [20-25]. An SMR of 2.1 (1.3–17.6) was applied to all ages for the portion of the population infected due to transfusion [26]. The number of active PWID and HCV prevalence among PWID was gathered through published studies [27-30] and divided by the total HCV-infected population to estimate percentage of all HCV infections that were among active PWID.

**Markov model —** The Markov model described here is an open-source model that is provided to academic and government researchers upon request. Modelers and epidemiologists in France, Greece, Australia, Egypt, Spain and Portugal have independently reviewed the model and provided feedback for modifications and updates. In addition, country experts in 59 countries continue to provide requests for updates to the model to enhance its functionality and algorithms. Since its inception in 2012 [31], the model has undergone over 80 revisions and updates.

The Markov (disease progression) model was constructed in Microsoft Excel® (Microsoft Corp., Redmond, WA) to quantify the annual size of the HCV-infected population by stage of liver disease over 1950–2050. The size and impact of the HCV-infected population prior to 1950 was considered negligible for the purposes of this analysis. Microsoft Excel was selected as a platform due to its transparency, availability and minimal need for operator training. The disease progression was modeled using the flow shown in the figure below and calculations shown in Equation 1.

The model started with the annual number of acute infections that progressed to chronic (viremic) HCV infection after accounting for spontaneous clearance of the virus. The methodology to calculate incidence is described below. The progression of these new cases was followed along with all chronic infections from prior years. Unless otherwise specified, the scope of the model was limited to viremic, HCV ribonucleic acid (RNA)-positive cases. Non-viremic cases (those exposed to the virus but spontaneously cleared the virus or were treated and cured) were not considered.

The number of new (incident) cases at each stage of disease was calculated annually by multiplying the annual progression rate times the prevalent population (by age and sex) in the previous stage, less cures and deaths. Thus, the annual number of new F2 cases was calculated by multiplying the prevalent population in F1 (by age and sex) less cures and deaths in the prevalent population in F1 times the F1–F2 progression rate.

The prevalent population at each stage of the disease was tracked by one-year age group and was aged (progressed to the next age group) annually. The progression rates were back-calculated using five-year age groups (as described below). In this model, the progression rate was assumed to be constant over the five-year age group. Thus, for ages 5–9, the F1–F2 progression rate was assumed to be constant.

The flow of the HCV disease progression model

Equation 1. Prevalent cases in stage of liver disease $\boldsymbol{x}$, at time $\boldsymbol{t}$, of sex $\boldsymbol{s}$, and age $\boldsymbol{a}$

$${\text{Prevalent} \text{cases}}_{\boldsymbol{x}\mathbf{,}\boldsymbol{t}\mathbf{,}\boldsymbol{s}\mathbf{,}\boldsymbol{a}}=\text{Prevalent cases}_{x,t-1,s,a-1,}\times\left( 1-d_{t-1,s,a-1} \right)\times\left( 1-l_{x,t-1,s,a-1} \right)\times\left( 1-p_{{x\to y}_{1},s,a-1} \right)\times\left( 1-p_{{x\to y}_{2},s,a-1} \right)\times\cdots\times\left( 1-p_{{x\to y}_{n},s,a-1} \right)\times\left( 1-c_{x,t-1} \right)\times\left( 1-s_{x,t-1} \right)+\text{New cases}_{x,t,s,a}$$

where:

$\boldsymbol{d}_{\boldsymbol{t,s,a}}$ is annual background mortality rate at time $t$, for sex $s$, at age $a$

$\boldsymbol{l}_{\boldsymbol{x,t,s,a}}$ is annual liver-related mortality rate for stage $x$, at time $t$, for sex $s$, at age $a$

$\boldsymbol{p}_{\boldsymbol{x\to y}_{\boldsymbol{1}}\boldsymbol{,s,a}}$, $\boldsymbol{p}_{\boldsymbol{x\to y}_{\boldsymbol{2}}\boldsymbol{,s,a}}$, …, $\boldsymbol{p}_{\boldsymbol{x\to y}_{\boldsymbol{n}}\boldsymbol{,s,a}}$ are annual progression rates from stage $x$ to $y_{1}$, stage $x$ to $y_{2}$, …, stage $x$ to $y_{n}$, respectively, for sex $s$, at age $a$

$\boldsymbol{c}_{\boldsymbol{x,t}}$ is annual cure rate for stage $x$, at time $t$, defined as

$$c_{x,t}=\frac{{\text{Total annual treatment}\text{s}}_{x,t}\times\text{SVR rate}_{t}}{\text{Total treatment-eligible cases}_{x,t-1}}$$

$\boldsymbol{s}_{\boldsymbol{x,t}}$ is annual liver transplantation rate for stage $x$, at time $t$, defined as

$$s_{x,t}=\frac{\text{Total liver transplantations}_{x,t}}{\text{Total liver transplant-eligible cases}_{x,t-1}}$$

$\text{New cases}_{\boldsymbol{x}\mathbf{,}\boldsymbol{t}\mathbf{,}\boldsymbol{s}\mathbf{,}\boldsymbol{a}}$ is the number of cases incident or progressing to stage $x$, at time $t$, for sex $s$, at age $a$.

**Progression rates —** The progression rates by age, sex and fibrosis score were back-calculated. Data from the UK were used for the percentage increase in progression rate by age and sex [32]. However, this study only reported progression from chronic HCV to moderate chronic HCV and from moderate chronic HCV to cirrhosis. These reported rates were modified using a meta-analysis of published work to calculate progression for F0, F1, F2, F3 and F4 [33]. Finally, the modified progression rates were adjusted to fit historical HCC incidence by age and sex in the U.S. [34] after adjusting for the portion of all HCC cases attributed to HCV infection [35]. The progression rates to end-stage liver disease and liver-related deaths were based on previously published rates. Insufficient data were available to develop predictable rates by age and sex. Thus, the same rate was applied for all ages and both sexes [36-38]. The table below lists all progression rates along with the uncertainty intervals.

HCV disease progression rates

| Back-calculated annual progression rates — Males | | | | | | | | | | | | | | | | | | |
| --- | --- | --- | --- | --- | --- | --- | --- | --- | --- | --- | --- | --- | --- | --- | --- | --- | --- | --- |
| Age group | **0–**  **4** | **5–**  **9** | **10–**  **14** | **15–**  **19** | **20–**  **24** | **25–**  **29** | **30–**  **34** | **35–**  **39** | **40–**  **44** | **45–**  **49** | **50–**  **54** | **55–**  **59** | **60–**  **64** | **65–**  **69** | **70–**  **74** | **75–**  **79** | **80–**  **84** | **85+** |
| F0 to F1 | 5.3% | 5.3% | 5.3% | 5.3% | 5.3% | 5.3% | 5.3% | 5.3% | 13.9% | 13.9% | 17.1% | 17.1% | 19.4% | 19.4% | 21.8% | 21.8% | 21.8% | 21.8% |
| Low | 3.1% | 3.1% | 3.1% | 3.1% | 3.1% | 3.1% | 3.1% | 3.1% | 8.2% | 8.2% | 10.1% | 10.1% | 11.4% | 11.4% | 12.8% | 12.8% | 12.8% | 12.8% |
| High | 8.1% | 8.1% | 8.1% | 8.1% | 8.1% | 8.1% | 8.1% | 8,1% | 21.3% | 21.3% | 26.2% | 26.2% | 29.7% | 29.7% | 33.4% | 33.4% | 33.4% | 33.4% |
| F1 to F2 | 3.4% | 3.4% | 3.4% | 3.4% | 3.4% | 3.4% | 3.4% | 3.4% | 9.1% | 9.1% | 11.2% | 11.2% | 12.7% | 12.7% | 14.3% | 14.3% | 14.3% | 14.3% |
| Low | 2.0% | 2.0% | 2.0% | 2.0% | 2.0% | 2.0% | 2.0% | 2.0% | 5.3% | 5.3% | 6.6% | 6.6% | 7.5% | 7.5% | 8.4% | 8.4% | 8.4% | 8.4% |
| High | 5.3% | 5.3% | 5.3% | 5.3% | 5.3% | 5.3% | 5.3% | 5.3% | 13.9% | 13.9% | 17.1% | 17.1% | 19.4% | 19.4% | 21.8% | 21.8% | 21.8% | 21.8% |
| F2 to F3 | 5.4% | 5.4% | 5.4% | 5.4% | 5.4% | 5.4% | 5.4% | 5.4% | 14.3% | 14.3% | 17.5% | 17.5% | 19.9% | 19.9% | 22.4% | 22.4% | 22.4% | 22.4% |
| Low | 3.2% | 3.2% | 3.2% | 3.2% | 3.2% | 3.2% | 3.2% | 3.2% | 8.4% | 8.4% | 10.3% | 10.3% | 11.7% | 11.7% | 13.2% | 13.2% | 13.2% | 13.2% |
| High | 8.3% | 8.3% | 8.3% | 8.3% | 8.3% | 8.3% | 8.3% | 8.3% | 21.8% | 21.8% | 26.9% | 26.9% | 30.5% | 30.5% | 34.3% | 34.3% | 34.3% | 34.3% |
| F3 to C Cirrhosis | 5.7% | 5.7% | 5.7% | 5.7% | 5.7% | 5.7% | 5.7% | 5.7% | 9.3% | 9.3% | 9.3% | 9.3% | 10.4% | 10.4% | 20.0% | 20.0% | 20.0% | 20.0% |
| Low | 3.3% | 3.3% | 3.3% | 3.3% | 3.3% | 3.3% | 3.3% | 3.3% | 5.3% | 5.3% | 5.3% | 5.3% | 6.0% | 6.0% | 11.4% | 11.4% | 11.4% | 11.4% |
| High | 10.8% | 10.8% | 10.8% | 10.8% | 10.8% | 10.8% | 10.8% | 10.8% | 17.7% | 17.7% | 17.7% | 17.7% | 19.8% | 19.8% | 38.1% | 38.1% | 38.1% | 38.1% |
| F3 to HCC | 0.2% | 0.2% | 0.2% | 0.2% | 0.2% | 0.2% | 0.2% | 0.2% | 0.2% | 0.2% | 0.2% | 0.2% | 0.2% | 0.2% | 0.2% | 0.2% | 0.2% | 0.2% |
| Low | 0.2% | 0.2% | 0.2% | 0.2% | 0.2% | 0.2% | 0.2% | 0.2% | 0.2% | 0.2% | 0.2% | 0.2% | 0.2% | 0.2% | 0.2% | 0.2% | 0.2% | 0.2% |
| High | 0.3% | 0.3% | 0.3% | 0.3% | 0.3% | 0.3% | 0.3% | 0.3% | 0.3% | 0.3% | 0.3% | 0.3% | 0.3% | 0.3% | 0.3% | 0.3% | 0.3% | 0.3% |
| C Cirrhosis to DCC | 3.0% | 3.0% | 3.0% | 3.0% | 3.0% | 3.0% | 3.0% | 3.0% | 3.0% | 3.0% | 3.0% | 3.0% | 3.0% | 3.0% | 3.0% | 3.0% | 3.0% | 3.0% |
| Low | 2.1% | 2.1% | 2.1% | 2.1% | 2.1% | 2.1% | 2.1% | 2.1% | 2.1% | 2.1% | 2.1% | 2.1% | 2.1% | 2.1% | 2.1% | 2.1% | 2.1% | 2.1% |
| High | 4.1% | 4.1% | 4.1% | 4.1% | 4.1% | 4.1% | 4.1% | 4.1% | 4.1% | 4.1% | 4.1% | 4.1% | 4.1% | 4.1% | 4.1% | 4.1% | 4.1% | 4.1% |
| C Cirrhosis to HCC | 3.6% | 3.6% | 3.6% | 3.6% | 3.6% | 3.6% | 3.6% | 3.6% | 3.6% | 3.6% | 3.6% | 3.6% | 3.6% | 3.6% | 3.6% | 3.6% | 3.6% | 3.6% |
| Low | 2.7% | 2.7% | 2.7% | 2.7% | 2.7% | 2.7% | 2.7% | 2.7% | 2.7% | 2.7% | 2.7% | 2.7% | 2.7% | 2.7% | 2.7% | 2.7% | 2.7% | 2.7% |
| High | 4.8% | 4.8% | 4.8% | 4.8% | 4.8% | 4.8% | 4.8% | 4.8% | 4.8% | 4.8% | 4.8% | 4.8% | 4.8% | 4.8% | 4.8% | 4.8% | 4.8% | 4.8% |
| DCC to Death | 20.0% | 20.0% | 20.0% | 20.0% | 20.0% | 20.0% | 20.0% | 20.0% | 20.0% | 20.0% | 20.0% | 20.0% | 20.0% | 20.0% | 20.0% | 20.0% | 20.0% | 20.0% |
| Low | 16.0% | 16.0% | 16.0% | 16.0% | 16.0% | 16.0% | 16.0% | 16.0% | 16.0% | 16.0% | 16.0% | 16.0% | 16.0% | 16.0% | 16.0% | 16.0% | 16.0% | 16.0% |
| High | 24.0% | 24.0% | 24.0% | 24.0% | 24.0% | 24.0% | 24.0% | 24.0% | 24.0% | 24.0% | 24.0% | 24.0% | 24.0% | 24.0% | 24.0% | 24.0% | 24.0% | 24.0% |
| HCC to Death (Year 1) | 70.7% | 70.7% | 70.7% | 70.7% | 70.7% | 70.7% | 70.7% | 70.7% | 70.7% | 70.7% | 70.7% | 70.7% | 70.7% | 70.7% | 70.7% | 70.7% | 70.7% | 70.7% |
| Low | 43.0% | 43.0% | 43.0% | 43.0% | 43.0% | 43.0% | 43.0% | 43.0% | 43.0% | 43.0% | 43.0% | 43.0% | 43.0% | 43.0% | 43.0% | 43.0% | 43.0% | 43.0% |
| High | 77.0% | 77.0% | 77.0% | 77.0% | 77.0% | 77.0% | 77.0% | 77.0% | 77.0% | 77.0% | 77.0% | 77.0% | 77.0% | 77.0% | 77.0% | 77.0% | 77.0% | 77.0% |
| HCC to Death (Sub Yrs) | 16.2% | 16.2% | 16.2% | 16.2% | 16.2% | 16.2% | 16.2% | 16.2% | 16.2% | 16.2% | 16.2% | 16.2% | 16.2% | 16.2% | 16.2% | 16.2% | 16.2% | 16.2% |
| Low | 11.0% | 11.0% | 11.0% | 11.0% | 11.0% | 11.0% | 11.0% | 11.0% | 11.0% | 11.0% | 11.0% | 11.0% | 11.0% | 11.0% | 11.0% | 11.0% | 11.0% | 11.0% |
| High | 23.0% | 23.0% | 23.0% | 23.0% | 23.0% | 23.0% | 23.0% | 23.0% | 23.0% | 23.0% | 23.0% | 23.0% | 23.0% | 23.0% | 23.0% | 23.0% | 23.0% | 23.0% |

| Back-calculated annual progression rates — Females | | | | | | | | | | | | | | | | | | |
| --- | --- | --- | --- | --- | --- | --- | --- | --- | --- | --- | --- | --- | --- | --- | --- | --- | --- | --- |
| Age group | **0–**  **4** | **5–**  **9** | **10–**  **14** | **15–**  **19** | **20–**  **24** | **25–**  **29** | **30–**  **34** | **35–**  **39** | **40–**  **44** | **45–**  **49** | **50–**  **54** | **55–**  **59** | **60–**  **64** | **65–**  **69** | **70–**  **74** | **75–**  **79** | **80–**  **84** | **85+** |
| F0 to F1 | 4.4% | 4.4% | 4.4% | 4.4% | 4.4% | 4.4% | 4.4% | 4.4% | 11.6% | 11.6% | 14.3% | 14.3% | 16.2% | 16.2% | 18.2% | 18.2% | 18.2% | 18.2% |
| Low | 2.6% | 2.6% | 2.6% | 2.6% | 2.6% | 2.6% | 2.6% | 2.6% | 6.8% | 6.8% | 8.4% | 8.4% | 9.5% | 9.5% | 10.7% | 10.7% | 10.7% | 10.7% |
| High | 6.7% | 6.7% | 6.7% | 6.7% | 6.7% | 6.7% | 6.7% | 6.7% | 17.7% | 17.7% | 21.8% | 21.8% | 24.8% | 24.8% | 27.8% | 27.8% | 27.8% | 27.8% |
| F1 to F2 | 2.9% | 2.9% | 2.9% | 2.9% | 2.9% | 2.9% | 2.9% | 2.9% | 7.6% | 7.6% | 9.3% | 9.3% | 10.6% | 10.6% | 11.9% | 11.9% | 11.9% | 11.9% |
| Low | 1.7% | 1.7% | 1.7% | 1.7% | 1.7% | 1.7% | 1.7% | 1.7% | 4.5% | 4.5% | 5.5% | 5.5% | 6.2% | 6.2% | 7.0% | 7.0% | 7.0% | 7.0% |
| High | 4.4% | 4.4% | 4.4% | 4.4% | 4.4% | 4.4% | 4.4% | 4.4% | 11.6% | 11.6% | 14.3% | 14.3% | 16.2% | 16.2% | 18.2% | 18.2% | 18.2% | 18.2% |
| F2 to F3 | 4.5% | 4.5% | 4.5% | 4.5% | 4.5% | 4.5% | 4.5% | 4.5% | 11.9% | 11.9% | 14.6% | 14.6% | 16.6% | 16.6% | 18.6% | 18.6% | 18.6% | 18.6% |
| Low | 2.6% | 2.6% | 2.6% | 2.6% | 2.6% | 2.6% | 2.6% | 2.6% | 7.0% | 7.0% | 8.6% | 8.6% | 9.8% | 9.8% | 11.0% | 11.0% | 11.0% | 11.0% |
| High | 6.9% | 6.9% | 6.9% | 6.9% | 6.9% | 6.9% | 6.9% | 6.9% | 18.2% | 18.2% | 22.4% | 22.4% | 25.4% | 25.4% | 28.6% | 28.6% | 28.6% | 28.6% |
| F3 to C Cirrhosis | 4.7% | 4.7% | 4.7% | 4.7% | 4.7% | 4.7% | 4.7% | 4.7% | 7.7% | 7.7% | 7.7% | 7.7% | 8.7% | 8.7% | 16.7% | 16.7% | 16.7% | 16.7% |
| Low | 2.7% | 2.7% | 2.7% | 2.7% | 2.7% | 2.7% | 2.7% | 2.7% | 4.4% | 4.4% | 4.4% | 4.4% | 5.0% | 5.0% | 9.5% | 9.5% | 9.5% | 9.5% |
| High | 9.0% | 9.0% | 9.0% | 9.0% | 9.0% | 9.0% | 9.0% | 9.0% | 14.7% | 14.7% | 14.7% | 14.7% | 16.5% | 16.5% | 31.8% | 31.8% | 31.8% | 31.8% |
| F3 to HCC | 0.2% | 0.2% | 0.2% | 0.2% | 0.2% | 0.2% | 0.2% | 0.2% | 0.2% | 0.2% | 0.2% | 0.2% | 0.2% | 0.2% | 0.2% | 0.2% | 0.2% | 0.2% |
| Low | 0.2% | 0.2% | 0.2% | 0.2% | 0.2% | 0.2% | 0.2% | 0.2% | 0.2% | 0.2% | 0.2% | 0.2% | 0.2% | 0.2% | 0.2% | 0.2% | 0.2% | 0.2% |
| High | 0.3% | 0.3% | 0.3% | 0.3% | 0.3% | 0.3% | 0.3% | 0.3% | 0.3% | 0.3% | 0.3% | 0.3% | 0.3% | 0.3% | 0.3% | 0.3% | 0.3% | 0.3% |
| C Cirrhosis to DCC | 3.0% | 3.0% | 3.0% | 3.0% | 3.0% | 3.0% | 3.0% | 3.0% | 3.0% | 3.0% | 3.0% | 3.0% | 3.0% | 3.0% | 3.0% | 3.0% | 3.0% | 3.0% |
| Low | 2.1% | 2.1% | 2.1% | 2.1% | 2.1% | 2.1% | 2.1% | 2.1% | 2.1% | 2.1% | 2.1% | 2.1% | 2.1% | 2.1% | 2.1% | 2.1% | 2.1% | 2.1% |
| High | 4.1% | 4.1% | 4.1% | 4.1% | 4.1% | 4.1% | 4.1% | 4.1% | 4.1% | 4.1% | 4.1% | 4.1% | 4.1% | 4.1% | 4.1% | 4.1% | 4.1% | 4.1% |
| C Cirrhosis to HCC | 3.6% | 3.6% | 3.6% | 3.6% | 3.6% | 3.6% | 3.6% | 3.6% | 3.6% | 3.6% | 3.6% | 3.6% | 3.6% | 3.6% | 3.6% | 3.6% | 3.6% | 3.6% |
| Low | 2.7% | 2.7% | 2.7% | 2.7% | 2.7% | 2.7% | 2.7% | 2.7% | 2.7% | 2.7% | 2.7% | 2.7% | 2.7% | 2.7% | 2.7% | 2.7% | 2.7% | 2.7% |
| High | 4.8% | 4.8% | 4.8% | 4.8% | 4.8% | 4.8% | 4.8% | 4.8% | 4.8% | 4.8% | 4.8% | 4.8% | 4.8% | 4.8% | 4.8% | 4.8% | 4.8% | 4.8% |
| DCC to Death | 20.0% | 20.0% | 20.0% | 20.0% | 20.0% | 20.0% | 20.0% | 20.0% | 20.0% | 20.0% | 20.0% | 20.0% | 20.0% | 20.0% | 20.0% | 20.0% | 20.0% | 20.0% |
| Low | 16.0% | 16.0% | 16.0% | 16.0% | 16.0% | 16.0% | 16.0% | 16.0% | 16.0% | 16.0% | 16.0% | 16.0% | 16.0% | 16.0% | 16.0% | 16.0% | 16.0% | 16.0% |
| High | 24.0% | 24.0% | 24.0% | 24.0% | 24.0% | 24.0% | 24.0% | 24.0% | 24.0% | 24.0% | 24.0% | 24.0% | 24.0% | 24.0% | 24.0% | 24.0% | 24.0% | 24.0% |
| HCC to Death (Year 1) | 70.7% | 70.7% | 70.7% | 70.7% | 70.7% | 70.7% | 70.7% | 70.7% | 70.7% | 70.7% | 70.7% | 70.7% | 70.7% | 70.7% | 70.7% | 70.7% | 70.7% | 70.7% |
| Low | 43.0% | 43.0% | 43.0% | 43.0% | 43.0% | 43.0% | 43.0% | 43.0% | 43.0% | 43.0% | 43.0% | 43.0% | 43.0% | 43.0% | 43.0% | 43.0% | 43.0% | 43.0% |
| High | 77.0% | 77.0% | 77.0% | 77.0% | 77.0% | 77.0% | 77.0% | 77.0% | 77.0% | 77.0% | 77.0% | 77.0% | 77.0% | 77.0% | 77.0% | 77.0% | 77.0% | 77.0% |
| HCC to Death (Sub Yrs) | 16.2% | 16.2% | 16.2% | 16.2% | 16.2% | 16.2% | 16.2% | 16.2% | 16.2% | 16.2% | 16.2% | 16.2% | 16.2% | 16.2% | 16.2% | 16.2% | 16.2% | 16.2% |
| Low | 11.0% | 11.0% | 11.0% | 11.0% | 11.0% | 11.0% | 11.0% | 11.0% | 11.0% | 11.0% | 11.0% | 11.0% | 11.0% | 11.0% | 11.0% | 11.0% | 11.0% | 11.0% |
| High | 23.0% | 23.0% | 23.0% | 23.0% | 23.0% | 23.0% | 23.0% | 23.0% | 23.0% | 23.0% | 23.0% | 23.0% | 23.0% | 23.0% | 23.0% | 23.0% | 23.0% | 23.0% |

C Cirrhosis, compensated cirrhosis; HCC, hepatocellular carcinoma; DCC, decompensated cirrhosis; Sub Yrs, subsequent years

**Incidence —** The following methodologies were used to estimate incidence in each country.

*Historical Incidence*

*Back-calculation of incidence —* A back-calculation methodology was used to estimate incidence by year. In this case, the prevalence of HCV in 1950 (who are still alive at the time of known prevalence) was assumed to be zero, and the same methodology as above was used to estimate the average annual number of new infections per year between 1950 and the year of known prevalence. The analysis was refined by developing a relative incidence curve with the 1950 relative incidence set to 1. The relative incidence was mapped based on the known risk factors and start of blood screening in the country. In approved models, these relative incidence curves were discussed at length with the expert panel in order to best estimate the historical “shape” of the epidemic relative to 1950. For example, in many counties the incidence of HCV was estimated to increase beginning around the 1960s or 1970s (relative to 1950), and then decrease in the 1990s as HCV screening tests became more prevalent in blood banks and transfusion centers. Incidence data on acute infections were also used to inform the incidence trends in the model.

The model was used to solve for a constant, times the annual relative incidence that resulted in the known prevalence after adjusting for mortality and cures. In this *calibration* step, the number of new infections shown in Equation 2 was calculated to fit the known prevalence in a given year *y*.

Equation 2. Total HCV infections in year *y*

${\text{Total HCV }\text{i}\text{nfections}}_{\text{ }\mathrm{Year}_{y}\text{ }}\text{=}\sum_{\text{t}\text{=1950}}^{\text{ }\text{y}} \left( \text{New infections}_{\text{t}} \text{– }\text{Spontaneous clearances}_{\text{t}}\text{– }\text{Deaths}_{\text{t}} \text{– }\text{Cures}_{\text{t}} \right)$

The annual incident cases were distributed by age and gender, and the modeled distribution was compared to the reported distribution. An iterative process of modifying the relative incidence curve and allocation by age was used to match the two curves and estimate the annual number of new infections by year.

*Current & Future Incidence*

The current incidence (after the known prevalence) was calculated by using the last year’s incidence and asking the Swiss experts if they expect the future prevalence to decline, stay the same or increase. The rate of growth or decline was also collected. This was then used in the model to calculate the minimum annual incidence per year to achieve the desired growth rate. It was assumed the number of new infections per year would stay constant in the future in the absence of better information.

**Validation of the Swiss model —** The modeled outputs for 1990–2013 were validated against empirical data. Incident liver cancer data were obtained from the National Institute for Cancer Epidemiology and Registration and adjusted for hepatocellular carcinoma (HCC) based on histology data from the Geneva Tumor Registry, as shown in the figure (a) below. Histological data were available for 52% of tumors, of which, approximately 91% were HCC. In the absence of better information, we assumed the remaining 48% of tumors had a similar histological profile and included a range to capture the significant associated uncertainty — 91% (0–100%) HCC. The percentage of HCC attributable to HCV infection was estimated to be 44.5% (range 43.3–53.3%) [10]. Model outcomes at a prevalence of 0.5% (situational analysis midpoint) and 0.8% (alternate estimate) are show in figure (b) below.

(a) (b)

1. Razavi H, Estes C, Pasini K, Gower E, Hindman S. HCV treatment rate in select European countries in 2004-2010. Journal of Hepatology. 2013;58:S22-S3.

2. Razavi H, Waked I, Sarrazin C, Myers RP, Idilman R, Calinas F, et al. The present and future disease burden of hepatitis C virus (HCV) infection with today's treatment paradigm. J Viral Hepat. 2014;21 Suppl 1:34-59. doi: 10.1111/jvh.12248 [doi].

3. Federal Statistical Office FSO Switzerland's population 2016. 2017 Contract No.: December 19.

4. Division UNDoEaSAP. World population prospects: The 2012 revision New York: United Nations; 2014 [updated 2014 Online version updates various data; cited 2014 6/18/2014]. Available from: <http://esa.un.org/unpd/wpp/index.htm>.

5. Zahnd C, Brezzi M, Bertisch B, Giudici F, Keiser O. Situation Analysis on hepatitis B and C in Switzerland [Online]. Federal Office of Public Health; 2017 [cited 2017 October 23]. Available from: [www.bag.admin.ch/situationsanalyse-hepatitis](file:///\\vmware-host\Shared%20Folders\Dropbox%20(CfDA)\CDA%20Library\Manuscripts\2018%20S%20(Mullhaupt)%20Swiss%20HCV%20Prev%20Update\www.bag.admin.ch\situationsanalyse-hepatitis).

6. Armstrong GL, Wasley A, Simard EP, McQuillan GM, Kuhnert WL, Alter MJ. The prevalence of hepatitis C virus infection in the United States, 1999 through 2002. Ann Intern Med. 2006;144(10):705-14. Epub 2006/05/17. PubMed PMID: 16702586.

7. Federal Office of Public Health. Swiss mandatory notifications for hepatitis C per canton, FOPH/CD/EPI/RIC 29.08.16. 2016.

8. IMS Health. IMS Health MIDAS Data 2013 [updated 1/1/2013]. Available from: <http://www.imshealth.com/>.

9. Bruggmann P, Berg T, Ovrehus AL, Moreno C, Brandao Mello CE, Roudot-Thoraval F, et al. Historical epidemiology of hepatitis C virus (HCV) in selected countries. J Viral Hepat. 2014;21 Suppl 1:5-33. doi: 10.1111/jvh.12247 [doi].

10. Vitali GC, Laurent A, Terraz S, Majno P, Buchs NC, Rubbia-Brandt L, et al. Minimally invasive surgery versus percutaneous radio frequency ablation for the treatment of single small (</=3 cm) hepatocellular carcinoma: a case-control study. Surg Endosc. 2016;30(6):2301-7. doi: 10.1007/s00464-015-4295-6. PubMed PMID: 26534770.

11. Federal Office of Public Health. Swiss mandatory notifications for hepatitis C per canton, FOPH/CD/EPI/RIC 29.08.2016. 2016 2016. Report No.

12. Hatzakis A, Chulanov V, Gadano AC, Bergin C, Ben-Ari Z, Mossong J, et al. The present and future disease burden of hepatitis C virus (HCV) infections with today's treatment paradigm - volume 2. J Viral Hepat. 2015;22 Suppl 1:26-45. doi: 10.1111/jvh.12351 [doi].

13. Sibley A, Han KH, Abourached A, Lesmana LA, Makara M, Jafri W, et al. The present and future disease burden of hepatitis C virus infections with today's treatment paradigm – volume 3. Journal of Viral Hepatitis. 2015;22:21-41. doi: 10.1111/jvh.12476.

14. Yang JD, Kim B, Sanderson SO, St Sauver JL, Yawn BP, Pedersen RA, et al. Hepatocellular carcinoma in Olmsted County, Minnesota, 1976-2008. Mayo Clin Proc. 2012;87(1):9-16. doi: S0025-6196(11)00004-8 [pii];10.1016/j.mayocp.2011.07.001 [doi].

15. Cejas NG, Villamil FG, Lendoire JC, Tagliafichi V, Lopez A, Krogh DH, et al. Improved waiting-list outcomes in Argentina after the adoption of a model for end-stage liver disease-based liver allocation policy. Liver Transpl. 2013;19(7):711-20. doi: 10.1002/lt.23665 [doi].

16. Sperl J, Frankova S, Trunecka P. [Liver transplantation for chronic hepatitis C, the importance of antiviral treatment]. 2013 2013. Report No.

17. Carmiel-Haggai M. [Two decades of liver transplantation in Israel]. Harefuah. 2012;151(12):679-83, 721.

18. Krawczyk M, Grat M, Barski K, Ligocka J, Antczak A, Kornasiewicz O, et al. 1000 liver transplantations at the Department of General, Transplant and Liver Surgery, Medical University of Warsaw--analysis of indications and results. Pol Przegl Chir. 2012;84(6):304-12. Epub 2012/07/31. doi: 10.2478/v10035-012-0051-y. PubMed PMID: 22842743.

19. Adam R, Karam V, Delvart V, O'Grady J, Mirza D, Klempnauer J, et al. Evolution of indications and results of liver transplantation in Europe. A report from the European Liver Transplant Registry (ELTR). J Hepatol. 2012;57(3):675-88. Epub 2012/05/23. doi: 10.1016/j.jhep.2012.04.015. PubMed PMID: 22609307.

20. Engstrom A, Adamsson C, Allebeck P, Rydberg U. Mortality in patients with substance abuse: A follow-up in Stockholm County, 1973-1984. Int J Addict. 1991;26(1):91-106.

21. Frischer M, Goldberg D, Rahman M, Berney L. Mortality and survival among a cohort of drug injectors in Glasgow, 1982-1994. Addiction. 1997;92(4):419-27.

22. Hickman M, Carnwath Z, Madden P, Farrell M, Rooney C, Ashcroft R, et al. Drug-related mortality and fatal overdose risk: Pilot cohort study of heroin users recruited from specialist drug treatment sites in London. J Urban Health. 2003;80(2):274-87. doi: 10.1093/jurban/jtg030 [doi].

23. Oppenheimer E, Tobutt C, Taylor C, Andrew T. Death and survival in a cohort of heroin addicts from London clinics: A 22-year follow-up study. Addiction. 1994;89(10):1299-308.

24. Perucci CA, Davoli M, Rapiti E, Abeni DD, Forastiere F. Mortality of intravenous drug users in Rome: A cohort study. Am J Public Health. 1991;81(10):1307-10.

25. Bjornaas MA, Bekken AS, Ojlert A, Haldorsen T, Jacobsen D, Rostrup M, et al. A 20-year prospective study of mortality and causes of death among hospitalized opioid addicts in Oslo. BMC Psychiatry. 2008;8:8. doi: 1471-244X-8-8 [pii];10.1186/1471-244X-8-8 [doi].

26. Kamper-Jorgensen M, Ahlgren M, Rostgaard K, Melbye M, Edgren G, Nyren O, et al. Survival after blood transfusion. Transfusion. 2008;48(12):2577-84. doi: TRF01881 [pii];10.1111/j.1537-2995.2008.01881.x [doi].

27. Aceijas C, Stimson GV, Hickman M, Rhodes T. Global overview of injecting drug use and HIV infection among injecting drug users. AIDS. 2004;18(17):2295-303. doi: 00002030-200411190-00010 [pii].

28. Nelson PK, Mathers BM, Cowie B, Hagan H, Des Jarlais D, Horyniak D, et al. Global epidemiology of hepatitis B and hepatitis C in people who inject drugs: Results of systematic reviews. The Lancet. 2011;378(9791):571-83.

29. Aceijas C, Rhodes T. Global estimates of prevalence of HCV infection among injecting drug users. Int J Drug Policy. 2007;18(5):352-8. doi: S0955-3959(07)00091-6 [pii];10.1016/j.drugpo.2007.04.004 [doi].

30. UNAIDS. THE GAP REPORT 2014: People who inject drugs. Online: Joint United Nations Programme on HIV/AIDS, 2014.

31. Razavi H, Elkhoury AC, Elbasha E, Estes C, Pasini K, Poynard T, et al. Chronic hepatitis C virus (HCV) disease burden and cost in the United States. Hepatology. 2013;57(6):2164-70. Epub 2013/01/03. doi: 10.1002/hep.26218. PubMed PMID: 23280550.

32. Harris RJ, Thomas B, Griffiths J, Costella A, Chapman R, Ramsay M, et al. Increased uptake and new therapies are needed to avert rising hepatitis C-related end stage liver disease in England: modelling the predicted impact of treatment under different scenarios. J Hepatol. 2014;61(3):530-7. Epub 2014/05/16. doi: 10.1016/j.jhep.2014.05.008. PubMed PMID: 24824282.

33. Thein HH, Yi Q, Dore GJ, Krahn MD. Natural history of hepatitis C virus infection in HIV-infected individuals and the impact of HIV in the era of highly active antiretroviral therapy: a meta-analysis. AIDS. 2008;22(15):1979-91.

34. Surveillance, Epidemiology, and End Results (SEER) Program Research Data (1973-2013) [Internet]. National Cancer Institute. 2016 [cited August 10th 2016]. Available from: [www.seer.cancer.gov](file:///\\vmware-host\Shared%20Folders\Dropbox%20(CfDA)\CDA%20Library\Manuscripts\2018%20S%20(Mullhaupt)%20Swiss%20HCV%20Prev%20Update\www.seer.cancer.gov).

35. Altekruse SF, Henley SJ, Cucinelli JE, McGlynn KA. Changing hepatocellular carcinoma incidence and liver cancer mortality rates in the United States. Am J Gastroenterol. 2014;109(4):542-53. doi: ajg201411 [pii];10.1038/ajg.2014.11 [doi].

36. Bernfort L, Sennfalt K, Reichard O. Cost-effectiveness of peginterferon alfa-2b in combination with ribavirin as initial treatment for chronic hepatitis C in Sweden. Scand J Infect Dis. 2006;38(6-7):497-505. doi: PK71701726364401 [pii];10.1080/00365540500532803 [doi].

37. Ries L, Young G, Keel G, Eisner M, Lin Y, Horner M. SEER survival monograph: Cancer survival among adults: U.S. SEER program, 1988-2001, patient and tumor characteristics. Bethesda, MD: National Cancer Institute, SEER Program; 2007.

38. Harris RJ, Thomas B, Griffiths J, Costella A, Chapman R, Ramsay M, et al. Increased uptake and new therapies are needed to avert rising hepatitis C-related end stage liver disease in England: Modelling the predicted impact of treatment under different scenarios. J Hepatol. 2014;61(3):530-7. doi: S0168-8278(14)00318-3 [pii];10.1016/j.jhep.2014.05.008 [doi].
