# Supplementary material for: Progress toward implementing the Swiss Hepatitis Strategy: Is HCV elimination possible by 2030?
Source: PLoS One. 2018 Dec 31;13(12):e0209374. doi: 10.1371/journal.pone.0209374 (PMC6312389; doi:10.1371/journal.pone.0209374)
Supplement: S2 File — (DOCX) [file pone.0209374.s003.docx]

# S2 File. Outcomes of the sensitivity and uncertainty analyses

Key drivers of uncertainty in prevalence (2017 and 2030)

*The labels refer to the high and low value of the variable under consideration.*

Disease burden model outcomes with 95% uncertainty intervals, by scenario, 2016–2030

1. Base 2017


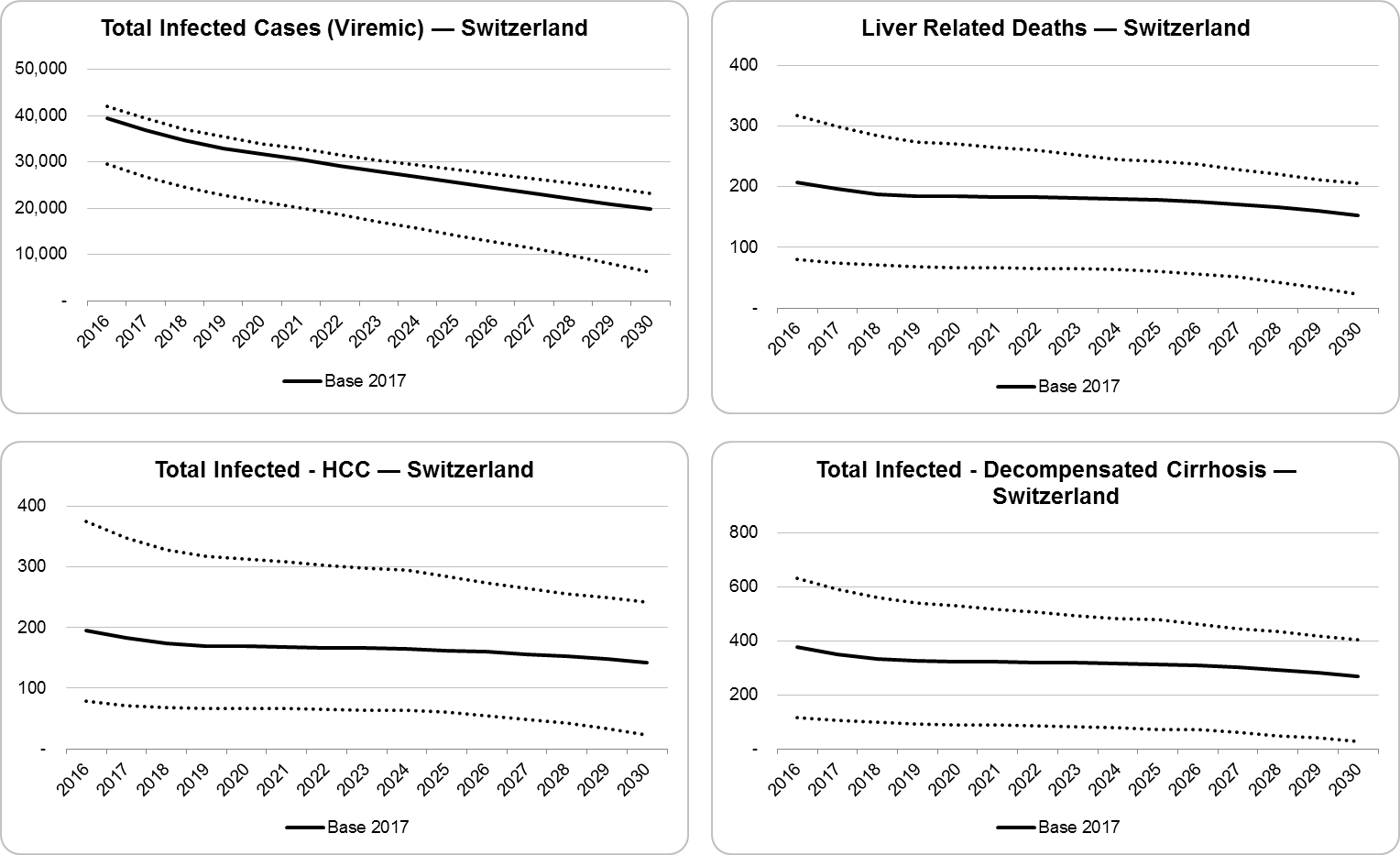


1. GHSS


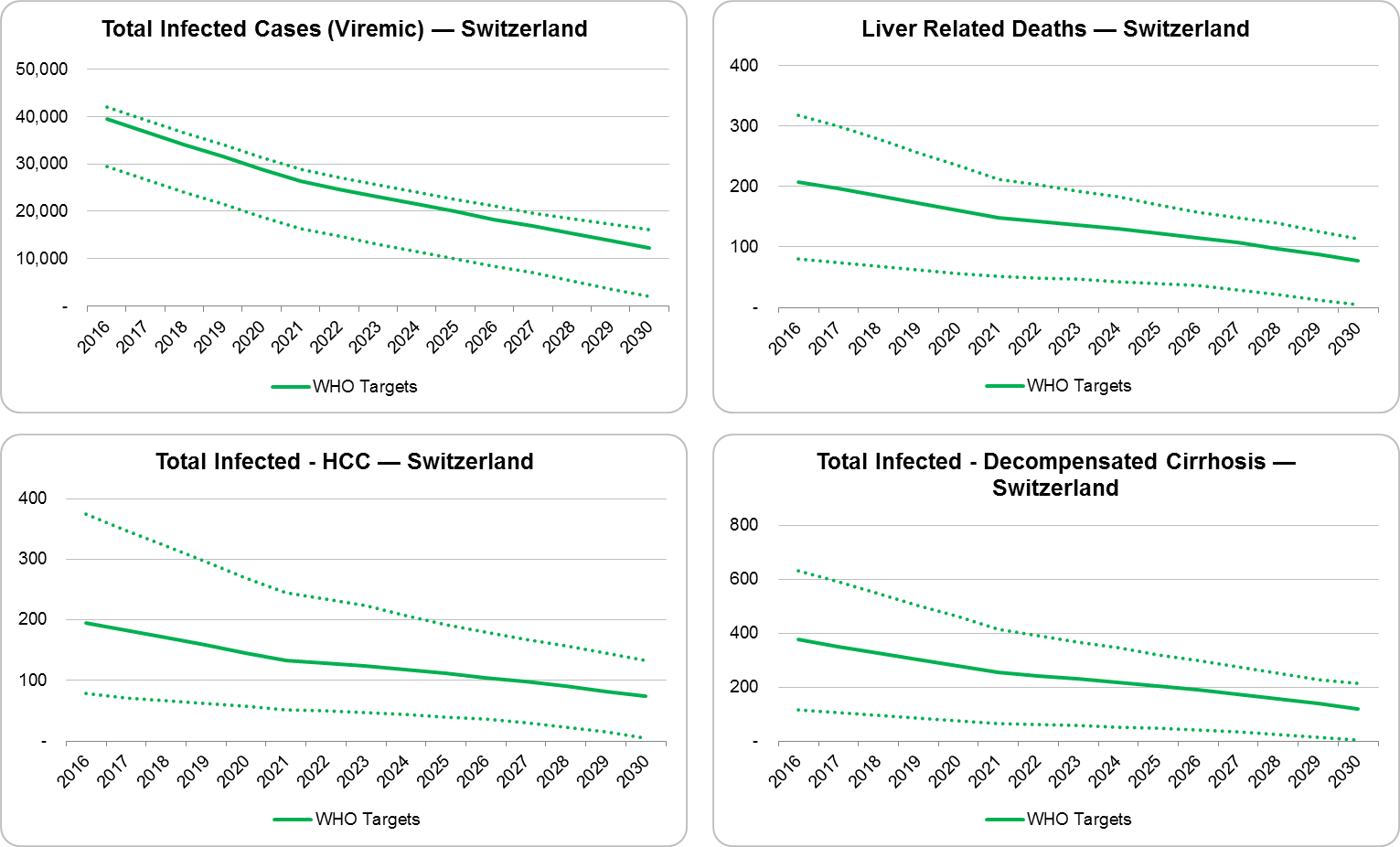


1. Swiss Hepatitis Strategy


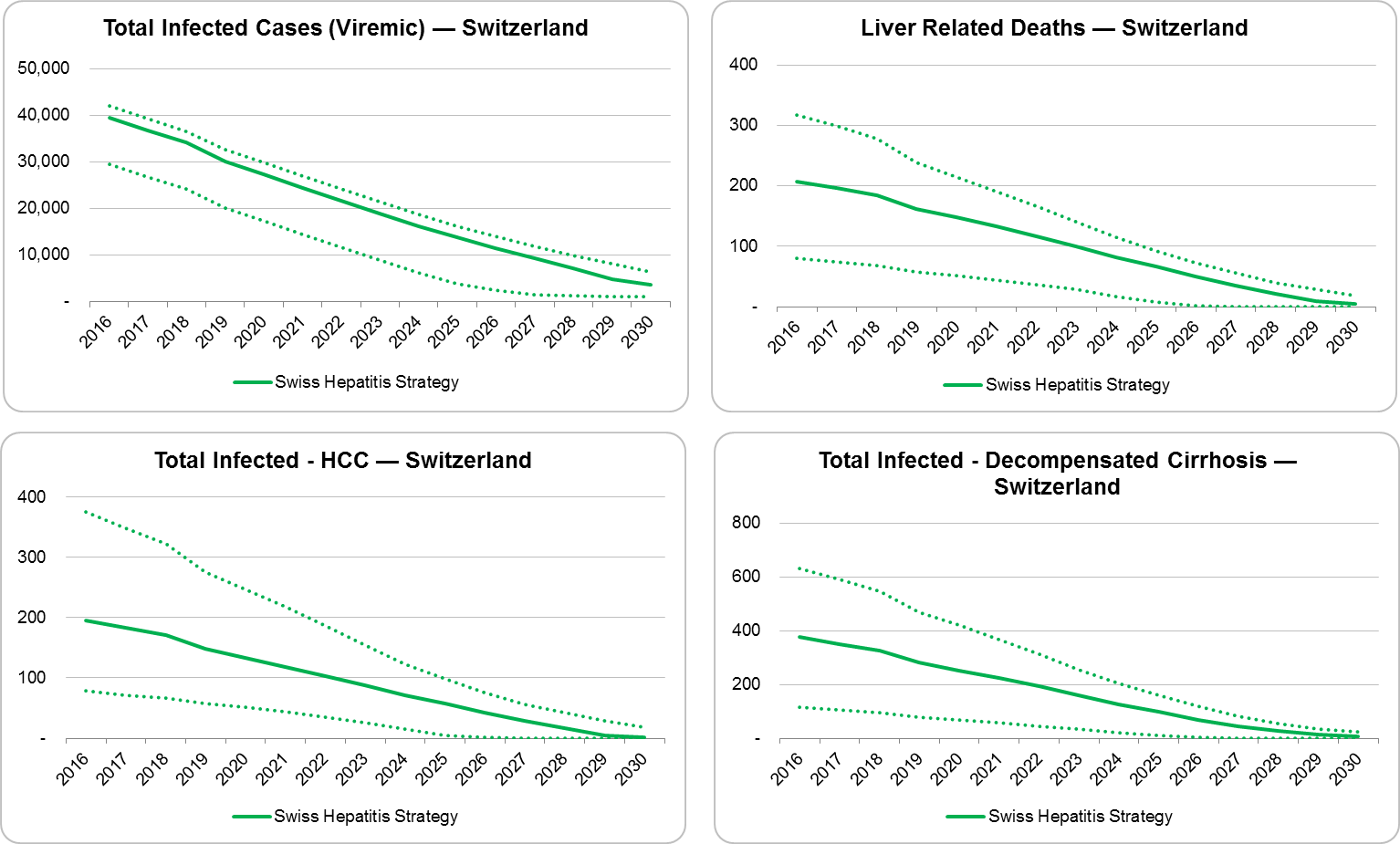


Annual number diagnosed and initiating treatment, 0.8% prevalence, under the elimination scenarios, 2016-2025.

| **Scenario Input** | **Scenario** | **Annual** | | | | | | | **Cumulative** |
| --- | --- | --- | --- | --- | --- | --- | --- | --- | --- |
|  |  | **2016** | **2017** | **2018** | **2019** | **2020** | **2022** | **≥2025** | **2018-2030** |
| Newly Diagnosed | GHSS | 1,100 | 1,100 | 1,100 | 1,100 | 4,000 | 4,000 | 3,200 | 41,400 |
|  | SHS | 1,100 | 1,100 | 1,100 | 4,500 | 4,500 | 4,500 | 4,500 | 38,500 |
| Initiating Treatment | GHSS | 2,000 | 3,000 | 3,000 | 3,000 | 4,500 | 4,500 | 3,300 | 48,300 |
|  | SHS | 2,000 | 3,000 | 3,000 | 4,900 | 4,900 | 4,900 | 4,900 | 61,800 |
